# Systemic Sclerosis Perturbs the Architecture of the Immunome

**DOI:** 10.3389/fimmu.2020.01602

**Published:** 2020-08-06

**Authors:** Bhairav Paleja, Andrea Hsiu Ling Low, Pavanish Kumar, Suzan Saidin, Ahmad Lajam, Sharifah Nur Hazirah, Camillus Chua, Lai Li Yun, Salvatore Albani

**Affiliations:** ^1^Translational Immunology Institute, Singhealth/Duke-NUS Academic Medical Centre, Singapore, Singapore; ^2^Department of Rheumatology and Immunology, Singapore General Hospital, Singapore, Singapore; ^3^Duke-NUS Medical School, Singapore, Singapore

**Keywords:** systemic sclerosis, MAIT, mass cytometry, immunome, chronic stimulation

## Abstract

Systemic sclerosis (SSc) is an autoimmune disease characterized by excessive fibrosis of skin and internal organs, and vascular dysfunction. Association of T and B cell subsets has been reported in SSc; however, there is lack of systematic studies of functional relations between immune cell subsets in this disease. This lack of mechanistic knowledge hampers targeted intervention. In the current study we sought to determine differential immune cell composition and their interactions in peripheral blood of SSc patients. Mononuclear cells from blood of SSc patients (*n* = 20) and healthy controls (*n* = 10) were analyzed by mass cytometry using a 36-marker (cell surface and intracellular) panel. Transcriptome analysis (m-RNA sequencing) was performed on sorted T and B cell subsets. Unsupervised clustering analysis revealed significant differences in the frequencies of T and B cell subsets in patients. Correlation network analysis highlighted an overall dysregulated immune architecture coupled with domination of inflammatory senescent T cell modules in SSc patients. Transcriptome analysis of sorted immune cells revealed an activated phenotype of CD4 and mucosal associated invariant T (MAIT) cells in patients, accompanied by increased expression of inhibitory molecules, reminiscent of phenotype exhibited by functionally adapted, exhausted T cells in response to chronic stimulation. Overall, this study provides an in-depth analysis of the systemic immunome in SSc, highlighting the potential pathogenic role of inflammation and chronic stimulation-mediated “functional adaptation” of immune cells.

## Introduction

Systemic sclerosis (SSc) is a multi-systemic autoimmune disease defined by excessive microvascular damage, dysregulated immune system, and increased deposition of extracellular matrix proteins leading to fibrosis of skin and internal organs (1). A lack of effective therapy is in part due to incomplete understanding of SSc pathogenesis, as well as inadequate mechanistic stratification of this heterogeneous disease. Traditional clinical subsetting according to the extent of skin thickening has shown that, in general, patients with limited cutaneous SSc (LcSSc) have a better prognosis than those with diffuse cutaneous SSc (DcSSc) (2). Although there are certain disease manifestations that differentiate these two groups, some of the severe organ manifestations, such as lung fibrosis, occur across groups and are the cause of significant mortality.

Crosstalk between stromal cells like fibroblasts and immune cells is considered as a major mechanism of disease pathogenesis and progression. However, the mechanisms responsible for the initiation of autoimmunity leading to fibrosis and the role of immune effector pathways in pathogenesis of SSc remain incompletely understood. A dysfunctional immune system that is evident in the periphery seems to be a major component of SSc pathogenesis, lending to the hope of being able to distill clinically and mechanistically meaningful signatures from peripheral blood. However, most of the knowledge is fragmented, underscoring the need for an integrated understanding of the architecture of the Immunome in SSc, thus potentially leading to therapies targeting the aberrant immune responses (3).

The objective of this study is to build a map of the immunome in SSc and to identify the perturbations that the disease causes by comparison with healthy controls. We employed for the purpose a combination of high dimensional technologies, namely, Cytometry by Time-of-Flight (CyTOF), for the immune architecture and next-generation RNA sequencing for the definition of the transcriptome in differential cell subsets identified by CyTOF. Our high-dimensional approach revealed disease-induced peculiarities in the immune architecture, with a polarization toward activated, pro-inflammatory, and senescent networks. More specifically, unsupervised clustering analysis revealed significant differences in the frequencies of T and B cell subsets in patients. Correlation network analysis highlighted an overall dysregulated immune architecture coupled with domination of inflammatory senescent T cell modules in SSc patients. Transcriptome analysis of sorted immune cells revealed an activated phenotype of CD4 and MAIT cells in patients, accompanied with increased expression of inhibitory molecules, reminiscent of phenotype exhibited by functionally adapted, exhausted T cells in response to chronic stimulation. Overall, this study provides an in-depth analysis of the systemic immunome in SSc, highlighting the potential pathogenic role of inflammation and chronic stimulation mediated “functional adaptation” of immune cells.

## Materials and Methods

### Patients

Blood samples were collected from 23 patients with SSc (from the Scleroderma Clinic, Department of Rheumatology and Immunology at Singapore General Hospital) and 10 age- and sex-matched healthy controls (HC). Patients fulfilling the 2013 American College of Rheumatology/European League Against Rheumatism criteria were included in this study. Of 23 patients enrolled, 21 patients (91%) were female. The mean age at disease onset from first non-Raynaud's phenomenon symptom was 41 years. Mean disease duration at time of blood collection was 5.2 years. Approximately half of the patients had LcSSc (*n* = 12) or DcSSc (*n* = 11). The frequency of anti-centromere antibodies and anti-topoisomerase-I antibodies was 3 and 12, respectively. The patients' clinical characteristics are summarized in Table 1. This study was approved by the Institutional Review Board of the Singapore General Hospital. All patients signed an informed consent to participate in the study.

**Table 1:** Patient clinical characteristics.

**Patient No**.	**Age**	**Gender**	**SSc subtype**	**Auto antibodies**	Disease duration^*^ (Years)	**Medication**	**Organ involvement**	Skin score^**^	**FVC; DLCO (% predicted)**
1	59	F	LcSSc	ACA	11.70	No	V, GI	10	92; 65
2	48	M	LcSSc	Topo-I	3.13	P, MMF	V, GI, ILD	0	54; 31
3	50	F	LcSSc	Topo-I	5.21	CYC	M, V, GI, ILD	3	47; 44
4	42	F	LcSSc	No	1.37	No	M, V, GI	0	68; 82
5	42	F	LcSSc	ACA	1.52	P	M, V, GI	5	91; 76
6	35	M	LcSSc	Topo-I	0.44	P	Cardiac, M, V, GI, ILD	6	60; 49
7	60	F	DcSSc	Topo-I	1.16	No	M, V, GI, ILD	10	53; 64
8	61	F	DcSSc	No	0.68	No	V, GI, ILD	27	52; 25
9	24	F	DcSSc	No	3.26	No	Cardiac, M, V, GI	2	76; 129
10	45	F	DcSSc	No	0.27	No	GI	8	100; 98
11	52	F	DcSSc	No	3.45	MTX	M, V, GI	2	79; 68
12	31	F	LcSSc	Topo-I	0.96	P, MMF	M, GI, ILD	6	48; 35
13	63	F	LcSSc	Topo-I	6.84	No	M, V, GI	1	81; 81
14	39	F	DcSSc	Topo-I	11.79	CYC	M, V, GI	17	96; 86
15	42	F	DcSSc	Topo-I	2.24	P, CYC	Cardiac, M, V, GI, ILD	12	58; 53
16	60	F	DcSSc	Topo-I	9.56	MMF	M, V, ILD	28	68; 48
17	22	F	DcSSc	Topo-I	1.00	No	M, V, GI, ILD	45	43; 48
18	47	F	DcSSc	No	3.91	P, MMF	M, V, GI, ILD	0	79; 52
19	62	F	LcSSc	No	10.93	No	M, V, GI, ILD	3	89; 68
20	55	F	LcSSc	No	26.53	No	M, V, GI	8	93; 70
21	33	F	LcSSc	ACA	2.43	P, MMF	M, V, GI	0	96; 101
22	59	F	LcSSc	Topo-I	10.45	MTX	M, V, GI, ILD	0	74; 65
23	36	F	DcSSc	Topo-I	0.27	MMF	M, GI	25	NA

* *Disease duration defined from first onset of non-Raynaud's phenomenon symptom*;

** *modified Rodnan skin score; F, female; M, male; LcSSc, limited cutaneous SSc; DcSSc, diffuse cutaneous SSc; ACA, Anti-centromere antibody; Topo-I, Anti-topoisomerase I antibody; P, Prednisolone; MMF, Mycophenolate mofetil; MTX, Methotrexate; CYC, Cyclophosphamide; GI, Gastrointestinal; ILD, Interstitial lung disease; M, Musculoskeletal; V, Vasculopathy; FVC, forced vital capacity; NA, not available*.

### Isolation of Peripheral Blood Mononuclear Cells

Peripheral blood was collected from all subjects in K2EDTA tubes and PBMCs were isolated by density gradient centrifugation using Ficoll-Paque Plus (GE Healthcare). Cells were resuspended in freezing medium containing 90% FBS and 10% DMSO, aliquoted, and stored in liquid nitrogen until use.

### Mass Cytometry Staining and Analysis

PBMCs were analyzed with 40 metal-conjugated antibodies specific for cell surface and intracellular antigens using CyTOF. Samples were thawed and left unstimulated or were stimulated with phorbol 12-myristate 13-acetate (PMA) and Ionomycin for 4 h. Brefeldin A and Monensin were added to culture in the last 3 h of incubation. The cells were stained with cisplatin to identify live cells. Next, a combination of four anti-CD45 antibodies were used as barcodes (4) to allow simultaneous sample processing and reduce technical variability. This was followed by incubation with metal-conjugated surface antibodies, fixation with 1.6% paraformaldehyde, permeabilization with 100% methanol, and intracellular metal-conjugated antibodies. Finally, the cells were labeled with iridium containing DNA intercalator. Samples were acquired on CyTOF 2 mass cytometer (Fluidigm).

EQ Four element calibration beads were used for signal normalization. Before downstream analysis, live cells were gated manually on event_length, ^195^Pt, and DNA (^191^Ir and ^193^Ir) and debarcoded using FlowJo software (Supplementary Figure 1). The debarcoded files were down-sampled to 5,000 cells each. These files were analyzed using an in-house developed software based on Barnes-Hut SNE non-linear dimension reduction algorithm followed by k-means clustering to identify immune cell clusters or “Nodes,” cells expressing similar expression of cell surface and intracellular markers (5). Heatmaps to identify phenotypes of significantly different nodes were plotted in R programming environment.

### Next-Generation Sequencing

Immune cell subsets identified as differentially expressed from CyTOF analysis (CD4^+^ T, CD8^+^ T, MAIT, and B cells) were sorted from PBMCs using antibodies against cell surface markers (CD3, CD4, CD8, CD19, CD14, CD161, TCR Vα7.2, and live/dead stain). PBMCs from six SSc patients and six healthy control were used for sorting. Based on the availability of cells from patients, three additional samples were used only for sorting and RNA-sequencing. Cells were sorted using a FACS Aria III sorter (BD Biosciences). After sorting, cells were washed, total RNA was isolated using a Picopure RNA-Isolation kit (Arcuturus, Ambion), and cDNA was generated using SMART Seq® v4 Ultra™ Low Input RNA Kit (Clontech), both according to the manufacturer's instructions. RNA isolated from sorted cells of human PBMCs had RNA integrity number (RIN) > 7. Illumina-ready libraries were prepared from cDNA using the Illumina Nextera XT DNA Library Prep Kit (Illumina, SD). The cDNA samples were evaluated after library prep using a bioanalyser. Next-generation sequencing (NGS) was performed at the NGS Platform of the Genome Institute of Singapore (GIS) using 2 × 151bp on a HiSeq 4000 platform.

### RNA-Sequencing Data Processing

Raw sequencing read data were evaluated using FastQC tool. Mean sequence quality (Phred score) of all the libraries was above 30. All the sequencing libraries passed the quality control test of FastQC. Raw RNA-sequencing reads were mapped to the human genome (Hg19) using STAR aligner (6) with default parameter. The counts of reads mapped over gene features were obtained using the featureCounts method of the Subread package (7). Fold change was calculated in the R statistical programming software. exactTest built in the edgeR package and recommended for two group tests was used for differential gene expression analysis. The RNA-sequencing data have been deposited in the NCBI BioProject repository under the accession code PRJNA639322.

### Network Analysis

Correlation Network analysis was performed as previously described (8). Briefly, proportion of each node was calculated for every sample and the correlation between nodes was determined for SSc and HC. To construct the network, nodes were connected if they had an absolute correlation coefficient >0.6. The network was visualized and analyzed using the igraph R package.

### Statistical Analysis

For CyTOF data, non-parametric unpaired Mann–Whitney tests (GraphPad Prism) were used to identify differential nodes between the two groups. *p* < 0.05 was considered statistically significant.

## Results

### Disease-Specific Alterations of Immune Cells in SSc

We designed a CyTOF panel composed of cell surface and intracellular markers (Supplementary Table 1) to comprehensively characterize peripheral immune cell composition in SSc patients and healthy controls. Initially, we characterized major immune cell lineages by assessing the frequency of T cells (CD3^+^), B cells (CD19^+^), monocytes (CD14^+^), and NK/ILCs (CD3^−^CD14^−^CD19^−^) in PBMCs from SSc patients and HC. No significant differences were found in total frequency of these subsets (Supplementary Figure 2). Further, we assessed the percentages of various T and B cell subsets in PBMCs from SSc patients and healthy controls. Expression of CD4 and CD8 was used to identify “conventional” T cells, whereas unconventional MAIT cells were identified as TCR Vα7.2^+^ CD161^+^ T cells (Supplementary Figure 3). MAIT cells as a frequency of total T cells were significantly reduced in SSc patients compared to healthy controls. In addition, frequencies of CD4^−^CD8^−^ T cells were also decreased in SSc patients. B cells were classified into naive, memory, and plasma blasts based on expression of CD19 and CD27 (Supplementary Figure 4). We found a decrease in memory B cell and a concomitant increase in naive B cells in patients.

**Figure 1:**
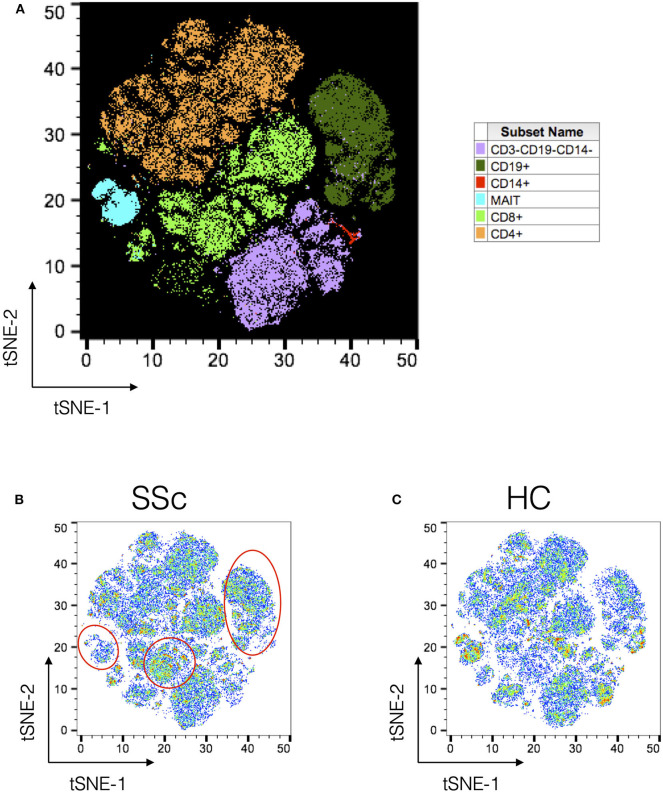
Unsupervised clustering reveals disease-specific alterations in PBMCs from systemic sclerosis patients. Unsupervised clustering analysis of mass cytometry data from PBMCs of SSc patients (*n* = 20) and healthy controls (*n* = 10). **(A)** tSNE maps for distribution of major immune subsets in SSc patients and healthy controls. All markers in the CyTOF panel were used for clustering analysis. **(B,C)** tSNE maps showing distribution of cells for SSc and HC, respectively. Marked regions (red circle/ovals) highlight changes in cell subsets in patients vs. healthy controls.

Next, PBMCs from SSc patients and healthy controls were analyzed using t-Distributed Stochastic Neighbor Embedding (tSNE) algorithm for dimension reduction, followed by clustering to identify nodes composed of similar cells. A comprehensive panel of antibodies, specific for lineage, activation, cytokines, trafficking, and differentiation, was designed and employed for the task. Figure 1A shows the tSNE plot of the distribution of all the major immune lineages in SSc patients and HC. Comparing similar plots for patients and healthy controls (Figures 1B,C, respectively) identified disease-specific alterations in immune cell subsets in SSc as evidenced from manual gating analysis.

### Architecture of Immunome Is Dysregulated in SSc

**Figure 2:**
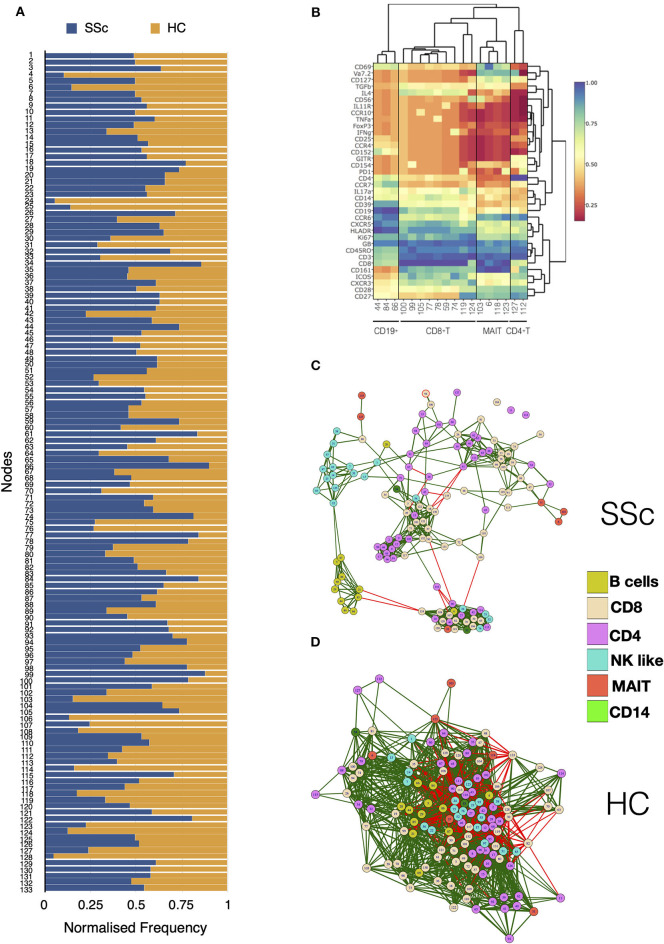
MAIT, CD4+, and CD8+ T cell subsets are affected in systemic sclerosis patients. Unsupervised clustering analysis of mass cytometry data from PBMCs of SSc patients (*n* = 20) and healthy controls (*n* = 10) was performed. **(A)** Stacked bar chart shows normalized frequency of all nodes identified by unsupervised clustering analysis. **(B)** Heatmap shows phenotype of nodes that were significantly (*t*-test) different between SSc patients and HC. Median expression of all markers for each node was determined, normalized, and plotted. **(C)** Correlation network for all the nodes identified by unsupervised clustering in PBMCs from SSc patients. **(D)** Correlation network for all the nodes identified by unsupervised clustering analysis in healthy controls. The color of the nodes identifies different cell types as indicated in the legend. Green and red lines indicate positive and negative correlation, respectively.

Unsupervised clustering analysis of CyTOF data from PBMCs of patients and HC identified a total of 133 nodes, with tight groupings of cells belonging to the same lineages. Comparison of frequencies of each node showed differences in immune subsets in SSc patients as compared to HC (Figure 2A). Statistical analysis revealed 18 nodes significantly different in SSc patients compared to HC (Supplementary Figure 5). This included nodes representing T cell (CD4^+^, CD8^+^, and MAIT) and B cell subsets (Figure 2B). This sort of analysis is very informative, but does not fully exploit the high dimensionality of CyTOF. Hence, we set off a relational analysis between different cell subsets, in order to define and understand if the architecture of the immunome in SSc could be a variation from the normal. To this end, we performed pairwise correlations between all nodes. Correlation values were used to define edges and create network between cellular nodes identified. Nodes were connected only if the absolute correlation values among them was >0.6. Network edges were color coded to identify positive (green) and negative (red) correlations, with edge width determining absolute correlation. Network properties (Supplementary Table 2) were calculated and analyzed to determine system-level immune cell communication. Modularity score is a measure of network structure and reveals the strength of division of network in modules. Higher modularity score indicates a network with dense connections between nodes within modules but sparse connections between nodes in different modules. SSc patients showed higher modularity (0.26, Figure 2C) of nodes as compared to healthy controls (0.047, Figure 2D). Furthermore, the number of negatively correlated edges was reduced in patients (2.24%) as compared to healthy controls (30.4%). Another network property, centralization score, is an indicator of dominance of nodes in overall network. A higher centralization score was observed in the patient's cellular network (0.19) compared to the HC network (0.031). To determine the identity of cell types in the different modules, we color coded each node based on their phenotype. We found highly correlated distinct modules of B cells and NK-like cells in SSc patients. In addition, tight modules of positively correlated CD4 and CD8 T cell subsets were evident in patients with such distinct clustering absent in immune networks from healthy controls. In summary, overall network analysis showed higher modularity, higher centralization score, lower negatively correlated nodes, and distinct cell-type-specific modules in cellular network from patients. These network properties are indicative of emergence of disease-specific interactions in immune subsets and gearing of functions centralized around dominating nodes.

### Specific Changes in Immunome of SSc Patients With and Without Interstitial Lung Disease

Next, we analyzed associations of immune cell numbers with clinical characteristics of SSc patients. ILD is the most common pulmonary complication in SSc and has the greatest impact on morbidity and mortality. We classified SSc samples into patients with ILD (*n* = 10) or no ILD (*n* = 10), as confirmed by high-resolution computed tomography of the thorax.

**Figure 3:**
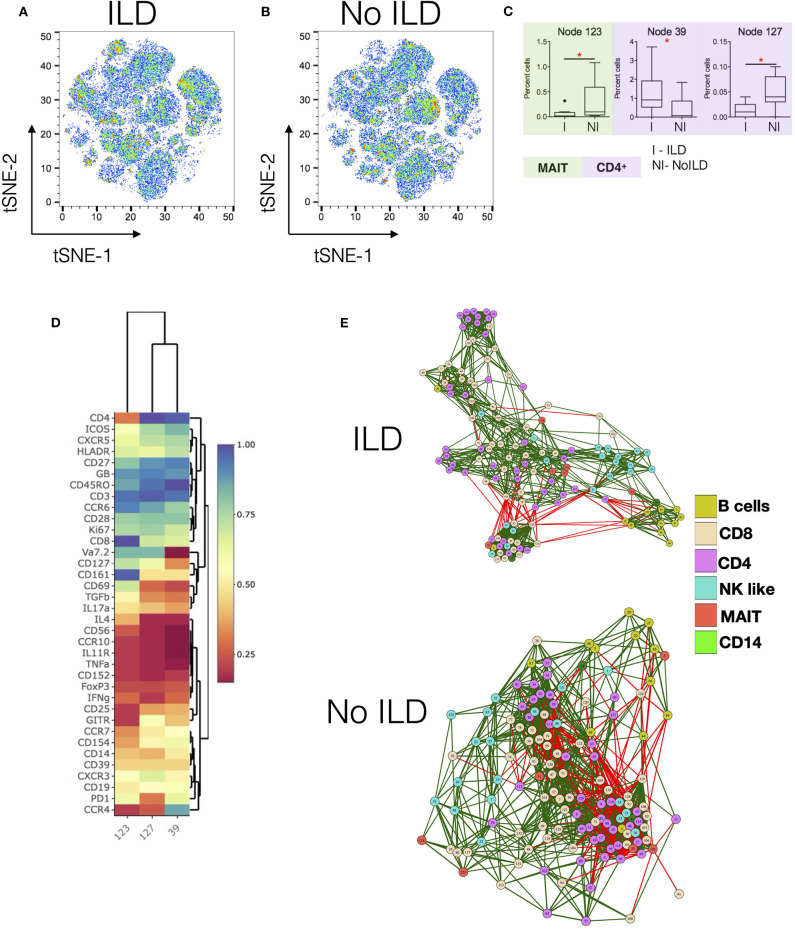
Specific changes in Immunome of SSc patients with and without interstitial lung disease. **(A,B)** tSNE maps for CyTOF analysis of SSc patients with interstitial lung disease (ILD, *n* = 10) and no ILD (*n* = 10), respectively. **(C)** Tukey plots showing frequencies of significantly different nodes in SSc patients. Plots are color coded to identify lineage of the node as per indicated. Unpaired *t*-test was performed for statistical analysis. **(D)** Heatmap shows phenotype of nodes that were significantly different between ILD and no-ILD patients. Heatmap shows normalized median marker expression. **(E)** Correlation networks showing relationship between different nodes in patients with ILD (upper panel) and No ILD (lower panel). The color of the nodes identifies different cell types as indicated in the legend. Green and red lines indicate positive and negative correlation, respectively. *p < 0.05.

Figures 3A,B show tSNE plots for ILD and no-ILD patients, respectively. Node 39 was found to be significantly increased in patients with ILD as compared to no ILD patients (Figure 3C). This node represented memory CD4^+^ T cells expressing CCR4 and ICOS (Figure 3D). Nodes 123 (MAIT cells) and 127 (CD4^+^ T cells), were significantly decreased in ILD patients as compared to no-ILD patients. Comparing the levels of these nodes in HC shows gradual decrease in frequency of these cell populations from HC > no ILD > ILD. Correlation network analysis of all nodes revealed subtle differences in the association of nodes in ILD vs. no-ILD patients (Figure 3E). ILD patients showed more modules of highly correlated nodes as compared to no-ILD patients. Nodes corresponding to CD4 and CD8 T cells were clustered together in modules seen exclusively in ILD patients.

Further analysis on immune cell composition in SSc patients was performed based on other clinical characteristics, including diffuse vs. limited cutaneous SSc or early vs. late disease. Increased B cells (cluster 44) and decreased MAIT (cluster 103) and CD4 T cells (cluster 112, cluster 127) were observed in DcSSc as compared to LcSSc patients (Supplementary Figure 6). However, these results were not statistically significant. Similarly, characterization of patients into early (<3 years) vs. late (>3 years) disease, showed no significant differences in frequencies of major immune cell lineages or different immune cell subsets (nodes).

### The Architecture of the Immunome in SSc Is Dominated by Pro-inflammatory Cells

**Figure 4:**
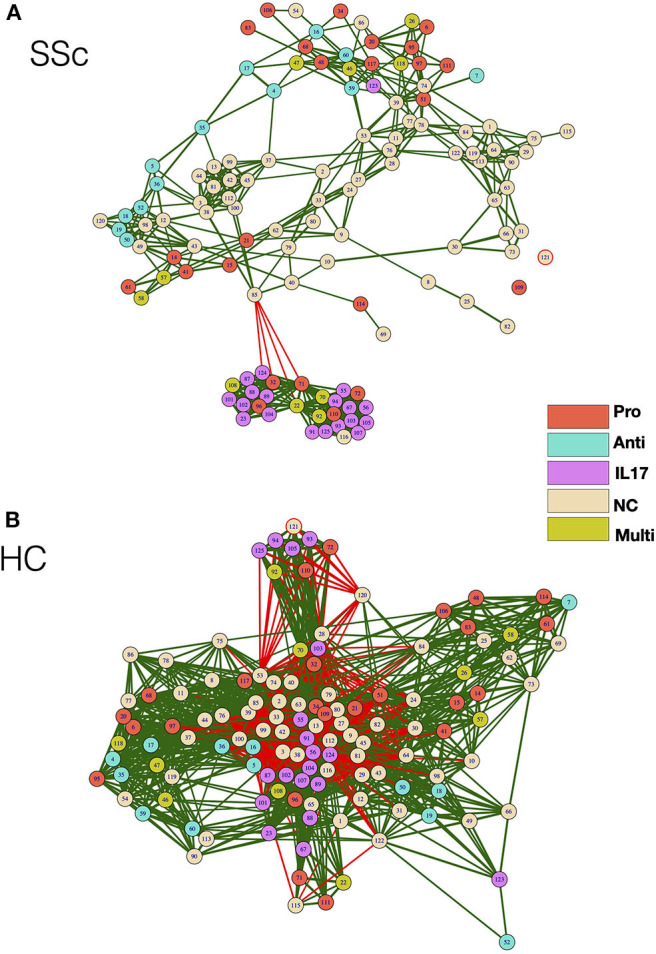
Correlation network shows enrichment of inflammatory cell subset modules in SSc patients. **(A,B)** Correlation networks showing interaction between different cytokine-producing cells from patients and healthy controls, respectively. The color of the nodes identifies the cytokine-producing capacity of cells in the particular node as indicated in the legend. Green and red lines indicate positive and negative correlation, respectively. The color of the nodes identifies the cytokine-producing capacity of cells in the particular node as indicated in the legend. Anti—Anti-inflammatory (IL-4-producing cells), Pro—Pro-inflammatory (IFN-γ, TNF-α, or both producing cells), IL-17—IL-17-producing cells, Multi—Multiple cytokines (cells producing a combination of cytokines), NC—No cytokine.

As cellular network analysis of unstimulated immune cells from patients revealed differential modular structure and intercellular connections as compared to healthy controls, we further investigated cellular networks in patients and healthy controls based on their functional status. Each node from the mass cytometry data of PMA/Ionomycin-stimulated cells was classified based on the ability of cells in the node to produce cytokines. Cells producing IFN-γ or TNF-α or both were classified as Pro-inflammatory, cells producing only IL-17 were classified as IL-17 producers, cells producing only IL-4 were classified as anti-inflammatory, cells producing any combination of the above combination were deemed as multi-cytokine producers. Cellular correlation network was then generated as previously described and nodes were color coded according to their functional responses. As observed for unstimulated cells, the correlation network of stimulated cells from patients vs. HC (Figure 4A) showed higher modularity (0.191 vs. 0.037, respectively), lower negative correlations (0.73 vs. 26.6%, respectively), and higher centralization score (0.334 vs. 0.039, respectively) as compared to healthy controls (Figure 4B). Interestingly, cells producing IL-17 formed a tight, highly correlated module in SSc patients but not in healthy controls. This, highly modular structure and grouping of IL-17-producing cells in patients is suggestive of the prevalence of inflammatory functions in SSc.

### Gene Signature Reveals Distinct Functional State of T Cells in SSc

**Figure 5:**
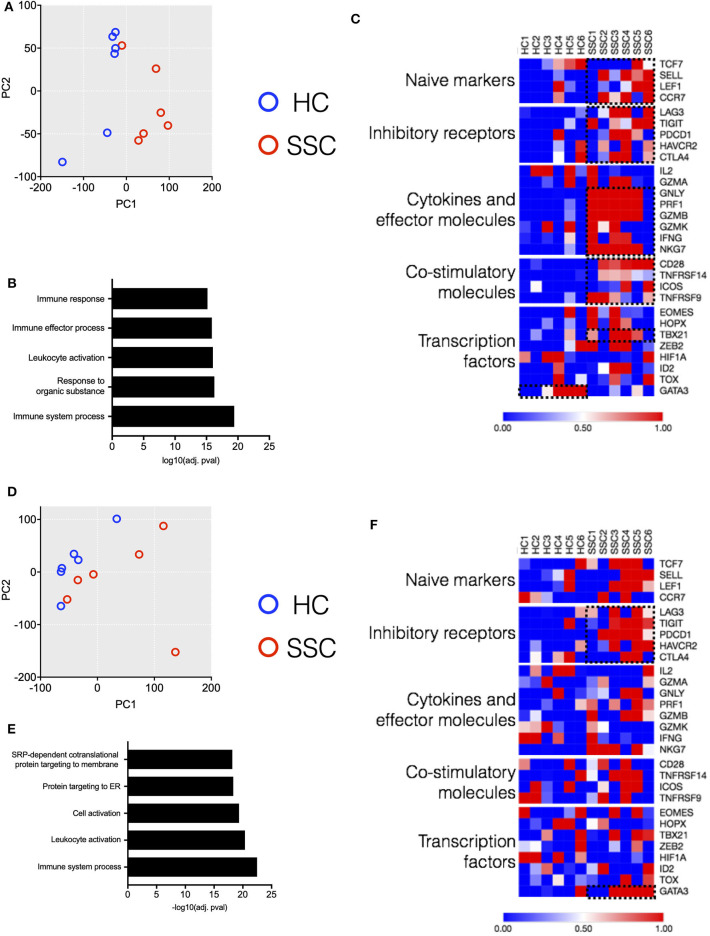
Transcriptome analysis of CD4 and MAIT cells reveals disease-associated clustering of biological samples. **(A,D)** Principal component analysis of CD4 T and MAIT cells, respectively, based on normalized read counts from RNA-seq data. Distinct separation of SSc patient samples (*n* = 6) and healthy controls (*n* = 6) is seen. **(B,E)** Enriched pathways unregulated in CD4 and MAIT cells, respectively, from SSc patients. **(C,F)** Normalized median expression of selected T cell function associated genes in CD4 and MAIT cells, respectively.

In order to better dissect the molecular mechanisms associated with the differences in the immune architecture in SSc patients, cell subsets that were differently represented in patients (i.e., CD4, CD8 T, MAIT, and B cells, Figures 1, 2) were sorted and subjected to transcriptome analysis by deep mRNA-sequencing (raw counts reported in Supplementary Table 3). We performed principal component analysis to visualize overall transcriptome changes in sorted cells from SSc patients and HC. T cells from patients segregated distinctly from healthy controls (Figures 5A,D). Indeed, differential gene expression showed distinct gene expression profile in all the T cell subsets analyzed, validating the finding by CyTOF that the immune mechanisms in SSc is functionally different from HC. In CD4 T cells, a total of 2266 genes were differentially regulated (1,082 genes upregulated and 1,184 genes downregulated) in SSc as compared to healthy controls (FDR cutoff <0.05 and minimum log2fold change = 2). In MAIT cells, a total of 762 genes were differentially regulated, 549 upregulated, and 213 downregulated in SSc as compared to healthy controls. In CD8 T cells, a total of 20 genes were differentially regulated, 10 upregulated and 10 downregulated in SSc as compared to healthy controls. In B cells, at the set cutoff, there were no genes differentially expressed. Enrichment analysis of differentially expressed genes revealed changes in various immune-related processes (Figures 5B,E) in CD4 and MAIT cells from patients.

Next, we delved into the mechanisms responsible for the differences in gene profiling. We analyzed from the RNAseq dataset how differently expressed genes affect T cell activation and functional status (9). CD4 T cells from patients showed an increased expression of genes related to cytokines and effector molecules (Figure 5C). Interestingly, these samples also exhibited higher expression of inhibitory receptor genes such as PDCD1, TIGIT, and LAG3 (Figure 5C). Similarly, MAIT cells from patients showed increased expression of inhibitory receptor genes (Figure 5F). The transcription factor GATA3 was highly expressed in MAIT cells from patients and not healthy controls. Conversely, CD4 T cells from patients showed increased expression of TBX21 as compared to healthy controls. Overall, transcriptome data revealed an activated phenotype of CD4 and MAIT cells in patients, though this was accompanied with increased expression of inhibitory/maturation molecules like PD1. Altogether, both the immunome and transcriptome profiles of T cell subsets eminent in SSc are reminiscent of “functionally adapted” senescent T cells in response to chronic stimulation, probably linking our findings to disease pathogenesis. We focused in particular on PD1 for its relevance in the cell responsiveness and homeostasis.

### SSc Is Associated With PD1 Expression and Terminal Differentiation of T Cells

**Figure 6:**
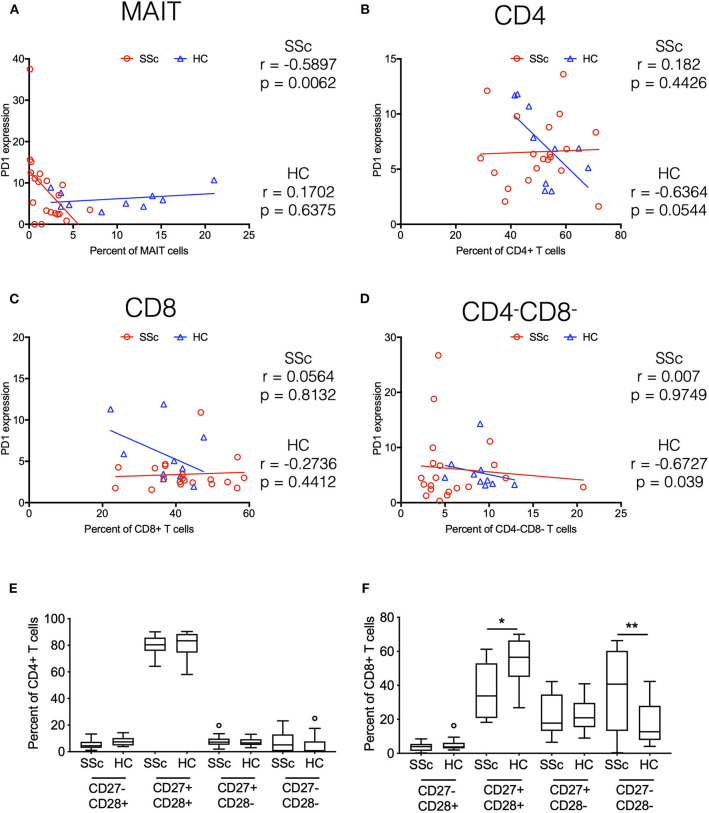
Systemic sclerosis is associated with PD1 expression and terminal differentiation of T cells. Correlation analysis between frequency of T cell subsets and PD-1 expression on the same cell subset. **(A–D)** Show MAIT cells, CD4+, CD8+, and CD4–CD8– T cells, respectively. Each point represents 1 sample, and a total of 20 SSc patients and 10 healthy controls are shown. Correlation analysis was performed using Spearman's rank correlation coefficient. **(E,F)** Proportion of CD27/CD28-expressing cells in total CD4 and CD8 T cells from patients and healthy controls. (*p < 0.05, **p < 0.01).

Indeed, in addition to its role in T cell exhaustion, PD1 has also been associated with expression on T cells under chronic stimulation (10). Moreover, elevated PD1 expression has been shown to be associated with attrition of MAIT cells in chronic infectious diseases (11). Our transcriptome data showed increased expression of PD1 on CD4 and MAIT cells from patients. To evaluate the role of increased PD1 expression in patients and its association with the frequency of various immune subsets in the periphery, we performed correlation analyses. PD1 expression on different T cell subsets (CD4^+^, CD8^+^, CD4^−^ CD8^−^, and MAIT cells) in our CyTOF data was determined by manual gating. Next, we investigated the association between PD1 expression on T cell subsets and frequency of the same cell type (Figures 6A–D). Results showed that frequency of MAIT cells in periphery in patients was inversely correlated with PD1 expression on MAIT cells (Figure 6A). Conversely, CD4^−^CD8^−^ T cells were inversely correlated with PD1 expression on CD4^−^CD8^−^ T cells in healthy controls but not in patients (Figure 6D).

Taken together, transcriptome and CyTOF data pointed toward an immune architecture dominated by chronic antigen-stimulated, senescent, pro-inflammatory rather than exhausted PD1^+^ T cells. To further ascertain this, we evaluated the differentiation state of CD4 and CD8 T cells. Based on the expression of co-stimulatory receptors CD27 and CD28, we observed an increased proportion of more differentiated CD27^−^CD28^−^ cells in CD8 T cells from SSc patients, with a concomitant decrease in CD27^+^CD28^+^ CD8 T cells (Figures 6E,F).

## Discussion

This study was conceived to address the specific hypothesis that there are aberrations of the architecture of the peripheral immunome in SSc which are mechanistically relevant. We employed for the purpose a combination of high dimensional technologies and analyzed the data in order to define spatially how the functional immune networks interlace.

Our approach departs from earlier efforts to understand the immune pathogenesis of SSc, which have focused on individual subsets utilizing conventional ways of analysis, providing a somewhat fragmented view of the immune landscape. There are contradictory reports on the frequencies of peripheral B cells in SSc patients. Indeed, the distribution of circulating B cells in SSc patients have shown to be increased (12), similar, or decreased (13). The frequencies of B cell subsets (naive, memory, and plasma blast) in our patients (data not shown) confirmed earlier reports (12) showing increased naive B cells and decreased memory B cells in the periphery of SSc patients. The decreased numbers of memory B cells have been attributed to an activated state and increased susceptibility to apoptosis (14).

MAIT cells represent a subset of unconventional T cells, characterized by invariant TCR repertoire and high expression of CD161. Since their identification recently, their role in infectious and autoimmune diseases is becoming increasingly evident. Our observation of reduced MAIT cells in the periphery of SSc patients mirrors the finding by Mekinian et al. (15). Similar findings have been reported in other conditions including autoimmune diseases like inflammatory bowel disease, Sjogren's syndrome, ankylosing spondylitis (AS), rheumatoid arthritis (RA), ulcerative colitis, and SLE (16–20) and infectious diseases like HIV and HCV (21, 22). Gracey et al. (18) reported a decreased MAIT cell frequency in AS and RA patients that likely reflected recruitment to site of inflammation such as gut or inflamed joint.

Newer high-dimensional technologies like mass cytometry and NGS make it possible to produce highly correlated data sets that capture the modularity and intercellular dynamics of an immune system. Such a holistic approach may enable the identification, at the systems level rather than mono-dimensionally, of the most relevant immune components responsible for pathogenesis of disease.

Our correlation network analysis revealed segregation of immune cell subsets into modules of correlated immune features and functions. We found a higher modularity, lower negatively correlated nodes, higher centralization score, and distinct cell-type-specific modules in SSc immune cell network compared to healthy controls, implying disease-specific interactions between immune subsets. Functional data also highlighted increased interplay between IL-17-producing cellular subsets in SSc patients compared to healthy controls. Our data are validated by reports that IL-17 and IL-17-producing cells are increased not only in the periphery but also in the skin in SSc (23–26). Yang et al. (27) showed also induction of fibroblast growth and increased collagen expression and protein secretion by IL-17. Our approach brings these observations into a functional and possibly pathogenic framework. The current evidences for the role of IL-17A as pro- or anti-fibrotic in SSc mouse models and patients have been inconclusive, with mice results indicating a pro-fibrotic role for IL-17 and conflicting conclusions in humans. Although further studies evaluating the role of IL-17 and IL-17-producing cells in SSc are warranted, overall, our data show that highly correlated immune cell subsets and functional modules are pivoting on IL-17 with obvious potential pathogenic and therapeutic implications.

Our approach also opened a new perspective on the immune pathogenies of the disease. In this study, we observed striking changes in transcripts of CD4 T and MAIT cells from SSc patients. Targeted analysis using a set of T cell-specific functional genes revealed increased expression of inhibitory-senescence receptors in CD4 and MAIT cells from SSc patients. Expression of co-inhibitory receptors, such as PD-1, LAG3, TIM-3, and TIGIT, on immune cells is crucial for the regulation of peripheral tolerance (28). Studies have reported increased expression of inhibitory receptors on immune cell subsets in various autoimmune diseases including SLE (29, 30), psoriatic arthritis (31), and rheumatoid arthritis (32). Fleury et al. (33) demonstrated increased expression and altered activity of inhibitory receptors in lymphocytes from SSc patients. Our transcriptome data highlighted changes in expression of such receptors on CD4 and MAIT cells in our patients. In addition, correlation analysis of mass cytometry data revealed an inverse correlation between PD1 expression and frequency of MAIT cells in SSc patients compared to healthy controls. In interpreting these data in a functional perspective, it must be underscored that expression of inhibitory receptors has been linked to reduced cytokine production by T cells in viral infections and cancer patients (34, 35). However, many recent studies have also shown that the expression of receptors like PD-1 does not correlate with lower functionality but rather differentiation state. True to this, our mass cytometry data showed an increased frequency of terminally differentiated subsets of CD27^−^CD28^−^ T cells in patients. The combination of our high-dimensionality data identified cell subsets reminiscent of functionally adapted, exhausted T cells in response to chronic stimulation.

The current study is limited by the number of samples analyzed and all the samples are of Asian ethnicity. In our study, we did not find statistical differences in immune cell composition in patients with different clinical characteristics, namely, DcSSc vs. LcSSc and early vs. late disease. This could either be due to smaller sample size analyzed in this study or indeed, there are no are differences in immunome of patients in the different disease subset. Moreover, there are balanced number of patients with ILD in each of DcSSc vs. LcSSc and early vs. late groups. Larger numbers would be needed to detect any differences in immunome expression beyond that is seen for ILD vs. no-ILD patients. Further studies on a larger cohort of patients are warranted to ascertain the possibility. Also, in the current study, EUSTAR revised activity index is not reported. It would be informative to analyze immunome in SSc patients in context with EUSTAR-AI.

Altogether, the combination of these data points toward a dominance in SSc of T cell subsets that are highly differentiated, chronically stimulated, and, as such, senescent, yet functional. Overall, this study delineates for the first time the architecture of systemic immunome in SSc, highlighting the roles of inflammation, and chronic stimulation-mediated terminal differentiation of immune cells. The knowledge distilled from this study has the dual translational valency of providing novel tools for monitoring and manipulating the deranged Immunome in SSc.

## Data Availability Statement

The RNA-sequencing data has been uploaded to NCBI BioProject repository under the accession code PRJNA639322. The other raw data supporting the conclusions of this article will be made available by the authors, without undue reservation, to any qualified researcher.

## Ethics Statement

The studies involving human participants were reviewed and approved by Institutional Review Board, Singapore General Hospital. The patients/participants provided their written informed consent to participate in this study.

## Author Contributions

BP performed the experiments, analyzed the data, and wrote the manuscript. PK performed bioinformatics and data analysis. SS, AL, SN, CC, and LL performed the experiments. AHLL recruited the patients and obtained the relevant blood samples. SA and AL conceived the study, analyzed the data, and wrote the manuscript. All authors contributed to the article and approved the submitted version.

## Conflict of Interest

The authors declare that the research was conducted in the absence of any commercial or financial relationships that could be construed as a potential conflict of interest.
