# Supplementary material for: Systemic Sclerosis Perturbs the Architecture of the Immunome
Source: Front Immunol. 2020 Aug 6;11:1602. doi: 10.3389/fimmu.2020.01602 (PMC7423974; doi:10.3389/fimmu.2020.01602)

Supplementary Table 1: CyTOF staining panel

Metal	Antibody	Clone	Vendor
**89**	CD45A	HI30	Fluidigm
**112/114**	CD14	TuK4	Lifetechnologies
**115**	CD45B	HI30	Biolegend
**139**	HLA-DR	L-243	Biolegend
**141**	CD39	A1	Biolegend
**142**	CD45RO	UCHL1	Biolegend
**143**	CD3	UCHT1	Biolegend
**144**	CD8	SK1	Biolegend
**145**	IL-4	8D4-8	Biolegend
**146**	CD28	CD28.2	Biolegend
**147**	PD-1	EH12.2H7	Biolegend
**148**	CD4	SK3	Biolegend
**149**	CCR10	6588-5	Biolegend
**150**	CD25	2A3	Biolegend
**151**	CD56	HCD56	Biolegend
**152**	TNF-a	Mab11	Biolegend
**153**	TGF-b	TW4-2F8	Biolegend
**154**	CD27	O323	Biolegend
**155**	CD152	BN13	BD Pharmingen
**156**	CD127	A019D5	Biolegend
**157**	CCR4	L291H4	Biolegend
**158**	CD154	24-31	Biolegend
**159**	CXCR5	J252D4	Biolegend
**160**	CD161	HP-3G10	Biolegend
**161**	CCR7	G043H7	Biolegend
**162**	FoxP3	PCH101	eBioscience
**163**	CXCR3	G025H7	Biolegend
**164**	GITR	621	Biolegend
**165**	IL11R	EPR5446	Biolegend
**166**	Ki67	20Raj1	eBioscience
**167**	ICOS	2D3	Biolegend
**168**	IFN-y	B27	Biolegend
**169**	IL-17A	BL168	Biolegend

Metal	Antibody	Clone	Vendor
**170**	CCR6	G034E3	Biolegend
**171**	CD45C	HI30	Biolegend
**172**	CD45D	HI30	Biolegend
**173**	GranzymeB	CLB-GB11	Abcam
**174**	CD19	HIB19	Biolegend
**175**	Va7.2	3C10	Biolegend
**176**	CD69	FN50	Biolegend
**191/193**	DNA		
**195**	Live/Dead		

Supplementary Table 2 : Network properties

Network property	Non stimulated		Stimulated	
	SSc	HC	SSc	HC
**Modularity Score**	0.26	0.047	0.191	0.037
**Network centralization**	0.19	0.031	0.334	0.039
**Network density**	0.065	0.24	0.070	0.232
**Avg. no. of neighbours**	9.2	34.59	8.72	28.8
**Negatively correlated edges**	13	659	4	480
**Positively correlated edges**	566	1503	541	1320
**% negatively correlated edges**	2.24	30.4	0.73	26.6

Supplementary Figure 1: Gating strategy employed

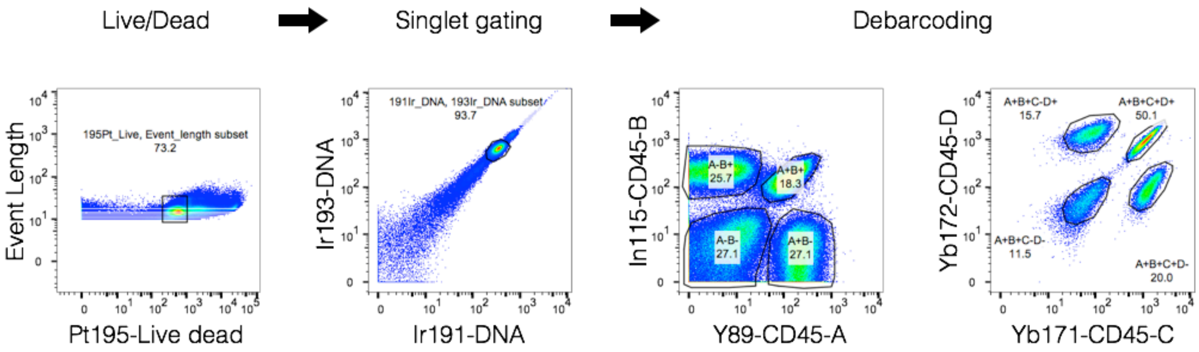

Supplementary Figure 2: Percentages of major immune cell lineages in peripheral blood

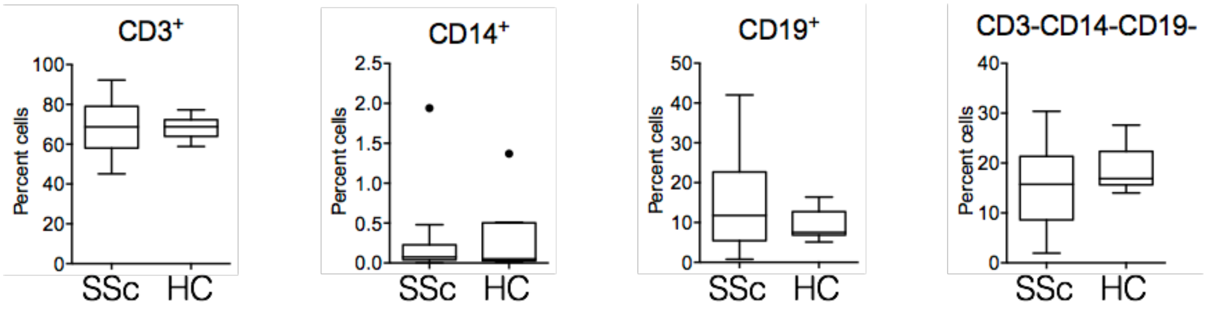

Supplementary Figure 3: Percentages of T cell subsets

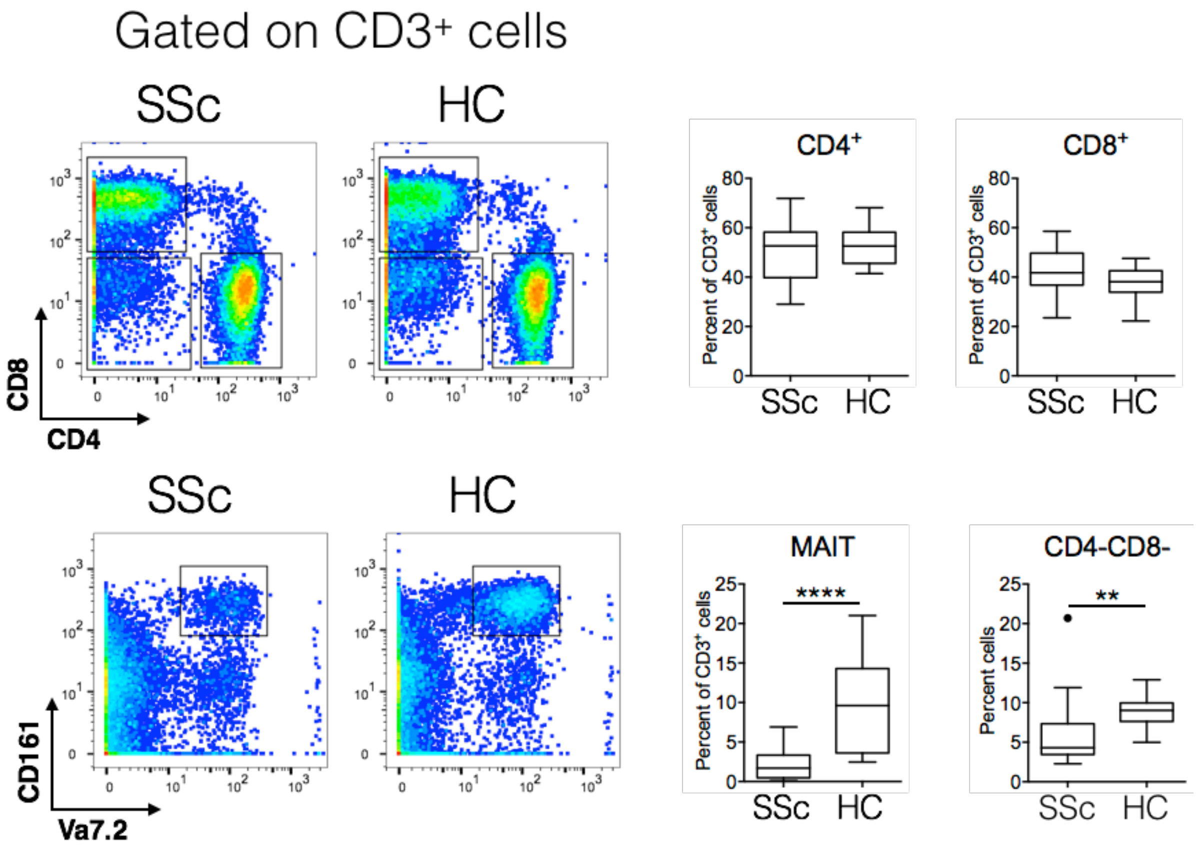

Supplementary Figure 4: Percentages of B cell subsets

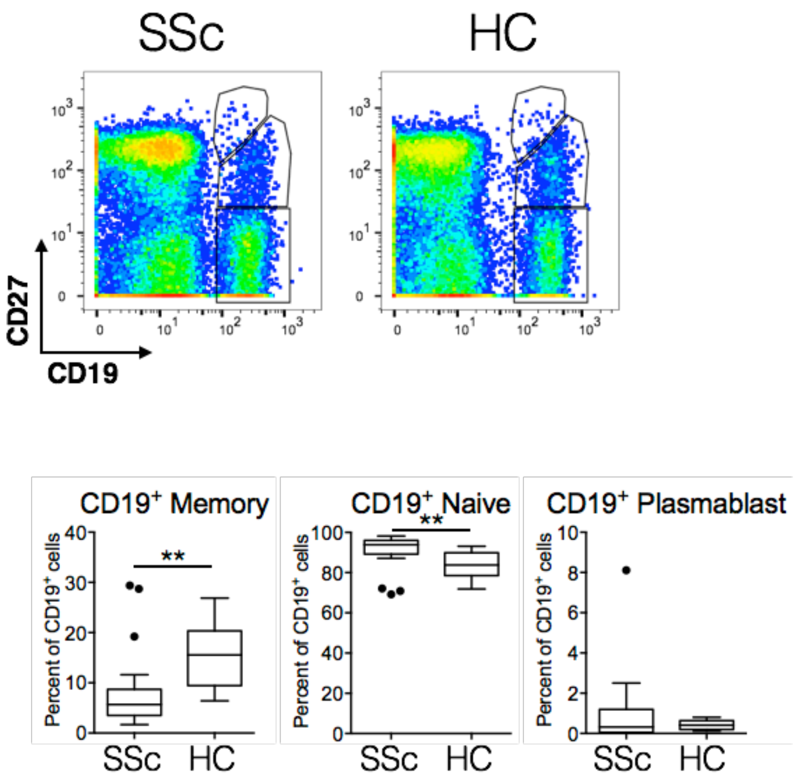

Supplementary Figure 5: Differentially expressed nodes in SSc patients

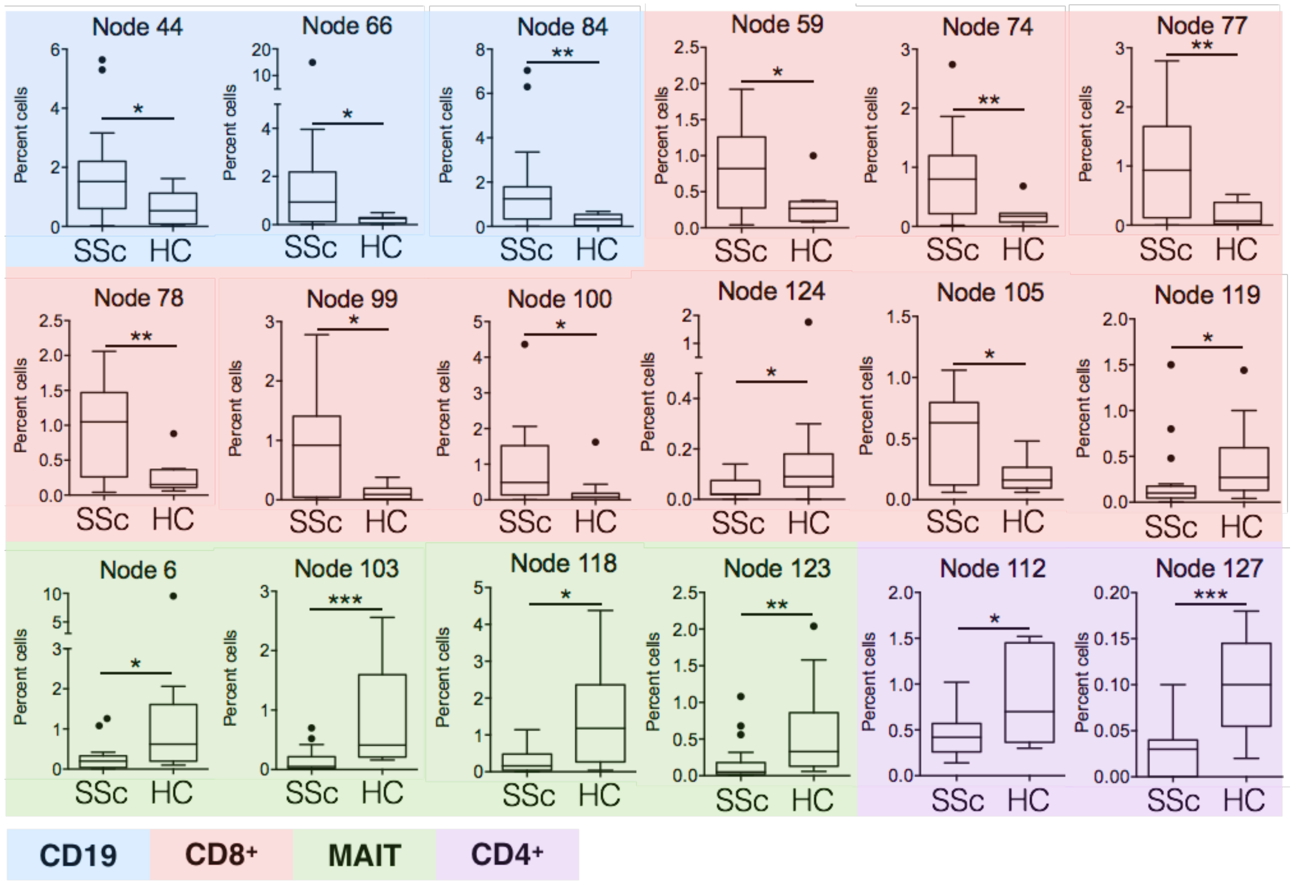

Supplementary Figure 6: Frequency of differential nodes in DcSSc vs LcSSc patients

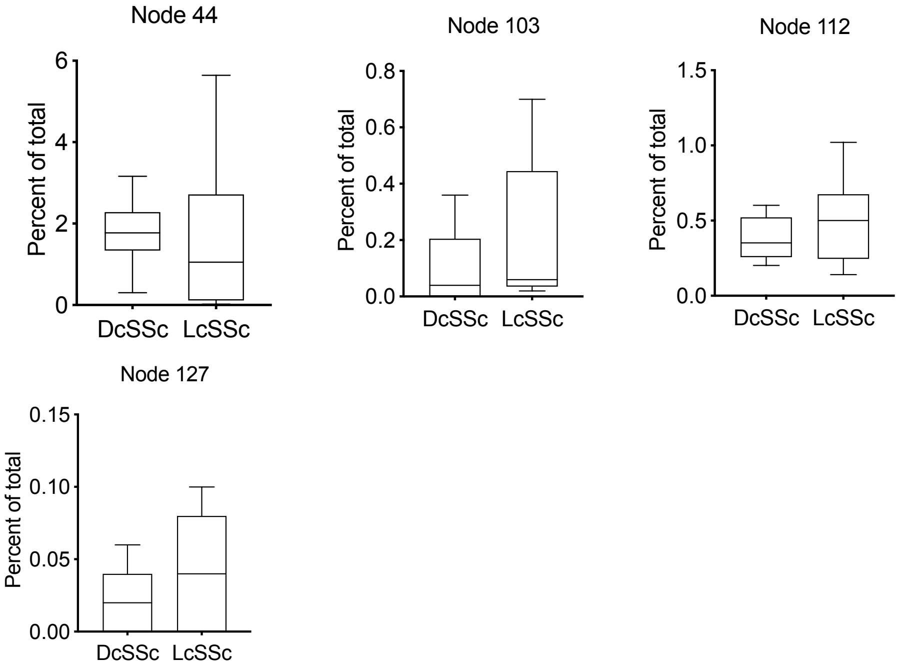

Supplement: Supplementary file 1 [file Data_Sheet_1.PDF]
